# MicroRNAs: The Role in Autoimmune Inflammation

**Published:** 2016

**Authors:** N. M. Baulina, O. G. Kulakova, O. O. Favorova

**Affiliations:** Pirogov Russian National Research Medical University, Ostrovityanova St., 1, Moscow, 117997, Russia; Russian Cardiology Research and Production Complex, 3-rd Cherepkovskay St., 15a, Moscow, 121552 , Russia

**Keywords:** microRNA, autoimmune inflammation, multiple sclerosis

## Abstract

MicroRNAs (miRNAs) are small non-coding RNA molecules that regulate gene expression at the post-transcriptional level through base-pairing predominantly with a 3’-untranslated region of target mRNA, followed by mRNA degradation or translational repression. Totally, miRNAs change, through a complex regulatory network, the expression of more than 60% of human genes. MiRNAs are key regulators of the immune response that affect maturation, proliferation, differentiation, and activation of immune cells, as well as antibody secretion and release of inflammatory mediators. Disruption of this regulation may lead to the development of various pathological conditions, including autoimmune inflammation. This review summarizes the data on biogenesis and the mechanisms of miRNA action. We discuss the role of miRNAs in the development and the action of the immune system, as well as in the development of an autoimmune inflammatory response. Special attention is given to the role of miRNAs in the autoimmune inflammation in multiple sclerosis, which is a serious socially significant disease of the central nervous system. Currently, a lot of research is focused on this problem.

## INTRODUCTION

Awareness of the fact that more than 80% of the genome has a specific biological function primarily associated with regulation of the expression of protein-coding genes became one of the most important results of the Encyclopedia of DNA Elements (ENCODE) project, which was aimed at deciphering the functional part of the genome. The most commonly identified functional elements were genes encoding various regulatory RNAs, including miRNAs (short, 19 to 24 nucleotide, single-stranded RNA molecules), which are key regulators of various biological processes at the post-transcriptional level [1].

The first miRNA (*lin-4*) was identified in the nematode* Caenorhabditis elegans *as early as in 1993, but only identification of the second miRNA (*let-7*) in *C. elegans *in 2000 provided the impetus for active investigation of miRNAs in vertebrates and invertebrates [2]. To date, miRNAs have been found in animals, plants, protists, and viruses [3]. The miRNA data are stored in a number of databases, including miRBase, microRNA. org, MicroRNAdb, miR2Disease, HMDD, and PhenomiR. According to the latest miRBase version (v21), a total of 35,828 mature miRNAs were identified in 223 species, with 2,588 mature miRNAs being identified in humans [4].

MiRNAs are highly conserved molecules. Evolutionary related miRNAs are combined into 239 different families whose members have highly homologous sequences and some common targets [5]. Recent studies have demonstrated that miRNAs are essential for the normal development of various physiological systems in organisms and maintenance of cell homeostasis, while a change in their expression and/or function is associated with the development of many pathological conditions in humans, including oncological, infectious, neurodegenerative, and autoimmune diseases [4]. In this review, we first discuss briefly the biogenesis and mechanism of action of miRNAs and then consider the involvement of miRNAs in the regulation of the immune system and the autoimmune inflammatory process, focusing on the participation of miRNAs in the development of multiple sclerosis (MS), which is a chronic autoimmune inflammatory disease of the central nervous system.

## BIOGENESIS AND MECHANISM OF ACTION OF miRNAs

Most miRNAs are encoded by genes located in the introns of protein-coding genes; miRNA genes can also be localized in exons, 5’- and 3’-untranslated gene regions, or intergenic regions [6].

**Fig. 1:**
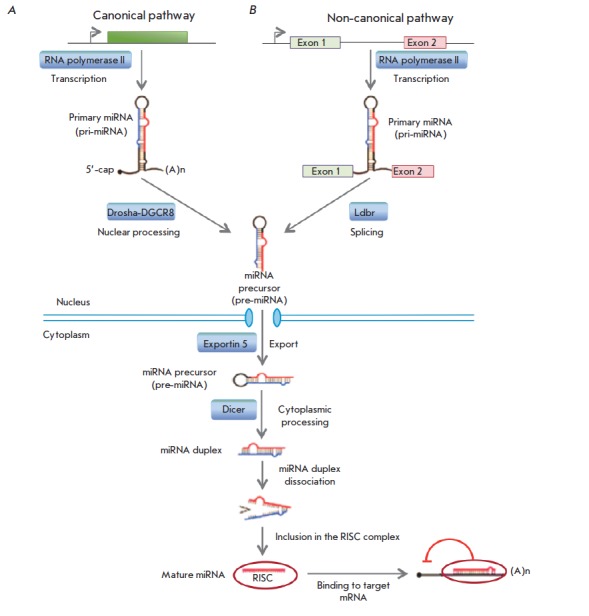
MiRNA biogenesis. A. The canonical pre-miRNA pathway produces pre-miRNAs through cleavage of pri-miRNA transcripts by the Drosha-DGCR8 microprocessor complex. B. The non-canonical pathway. Mirtrons are spliced and debranched by the Ldbr enzyme, after which they fold into pre-miRNA hairpins. Then, the pathways merge. The green box indicates a miRNA gene; exons 1 and 2 are exons of the host gene encoding intronic miRNA.

MiRNA genes are transcribed in the nucleus, primarily by RNA polymerase II, as a primary miRNA (pri-miRNA), which is a long transcript (from a few hundred to tens of thousands of nucleotides) (*Fig. 1*). The primary miRNA is then converted into a miRNA precursor (pre-miRNA) by the Drosha-DGCR8 microprocessor complex (canonical pathway) [6]. There are also several other non-canonical pathways of premiRNA production, one of which is the formation of a pre-miRNA during splicing of short hairpin introns (mirtrons), followed by cleaving of pre-miRNA by the Ldbr protein [7]. Then, the miRNA biogenesis pathways merge, and the pre-miRNA is processed in the cytoplasm by the Dicer enzyme (RNase III) to form a miRNA duplex, with one of the duplex chains being involved in the formation of the RNA-induced silencing complex (RISC) (*Fig. 1*).

A small, 6–8 nucleotide, miRNA fragment, the seed region, is critical for miRNA binding to the target mRNA within the RISC complex. The degree of complementarity between this miRNA fragment and the target mRNA largely determines the mechanism of gene expression regulation. Complete complementary binding between miRNA and mRNA leads to cleavage and degradation of mRNA. In the case of incomplete complementarity between miRNA and a target mRNA, mRNA translation is inhibited at the stage of initiation or elongation, and the mRNA is destabilized due to cleavage of a polyA sequence and is transferred to processing bodies. MiRNA can act at the transcriptional level through regulation of chromatin reorganization [9]. In most cases, miRNAs reduce the expression level of a target mRNA; but in some cases, binding of miRNAs to certain protein complexes can increase expression of target genes via direct or indirect mechanisms [10].

At present, miRNAs are known to function not only inside the cells, but are also capabale of being secreted into the bloodstream and affect other animal cells. In blood, an extracellular mature miRNA (90–99%) primarily associates with proteins of the AGO family [11]. In addition, pre-miRNA can be secreted into the bloodstream within exosomes and/or multivesicular bodies. Exosomes, in turn, can be captured by recipient cells (including other cell types), and the pre-miRNA in the cytoplasm of recipient cells is processed into a mature miRNA. MiRNA can also be released from cells during apoptosis [12].

To denote the gene encoding miRNA, its precursor, and a mature miRNA molecule, there is a special nomenclature, but it has not yet become conventional. For example, the gene encoding miRNA is denoted by two abbreviations: mir or *MIR*, e.g., mir142 or *MIR*142. The abbreviation “mir” is also used to denote a primary miRNA and pre-miRNA, while a mature miRNA is called “miR”. A certain species of miR origin is designated with a three-letter prefix: “hsa” means a human (*Homo sapiens*) miR, and “rno” means a rat (*Rattus norvegicus*) miR; e.g., hsa-miR-367 or rno-miR-1, respectively. Groups of closely related miRNAs having a similar sequence are combined into families designated by numbers (e.g., miR-33). Within the same family, individual miRNAs are annotated with an additional one-letter suffix; e.g., hsa-miR-451a and hsa-miR-451b. Pre-miRNAs that give rise to identical mature miRNAs but are encoded in different genome regions are denoted with an additional dash-number suffix; e.g., hsa-mir-121-1 and hsa-mir-121-2 precursors give the same mature miRNA hsa-miR-121. The miRNA duplex strand that preferentially binds to the target mRNA (also called the guide strand) is designated as, e.g., miR- 56, and the complementary unstable (passenger) chain is denoted by an asterisk (e.g., miR-56*). If data on the functional activity of miRNA duplex strands are missing, the pre-miRNA end related to the miRNA duplex strand resulting from processing is indicated, e.g., miR- 142-5p (5’-end of pre-miRNA) and miR-142-3p (3’-end of pre-miRNA).

**Fig. 2:**
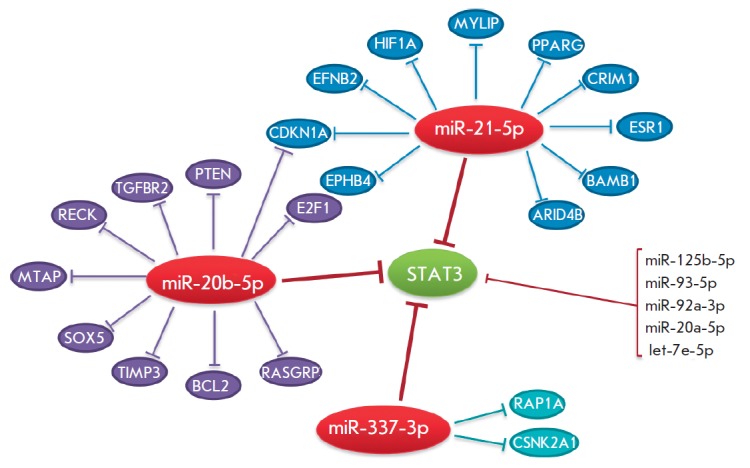
Redundancy and pleiotropy of the miRNA regulatory system. The STAT3 gene (green oval) encodes a transcriptional factor. Red ovals are miRNAs downregulating the STAT3 gene expression. Each of the miRNAs inhibits expression of other target mRNAs (blue, violet, and light blue ovals). Additional miRNAs that may affect the STAT3 gene expression are listed on the right-hand side. The regulatory network was simulated using the Mirtarbase database (http://mirtarbase.mbc.nctu.edu.tw/index.php).

Like cytokine action, the miRNA function is characterized by degeneracy (redundancy) and pleiotropy; i.e. the expression level of one mRNA can be regulated by many miRNAs, and one miRNA binds to many target mRNAs, which results in the formation of a complex regulatory network (*Fig. 2*). Thus, a change in the expression of one miRNA may lead to changes in the expression profile of many target mRNAs: however, this effect for each individual mRNA will also depend on the influence of other miRNAs.

Redundancy of the miRNA system may affect, in total, expression of about 60% of the genes in the organism [13]. It should be noted that the expression level of miRNA genes, like that of protein-coding genes, can be regulated at the epigenetic level, during transcription, processing, and nuclear export, as well as being controlled by the miRNA degradation level [14]. The spectrum of organism miRNAs directly depends on the complexity of the organism structure. In addition, miRNA expression is tissue-specific and ontologically oriented. Thus, as our understanding grows, it becomes increasingly clear that the miRNA network is an essential and evolutionarily ancient component of the system of gene expression regulation.

## INVOLVMENT OF miRNAs IN THE DEVELOPMENT AND FUNCTION OF THE IMMUNE SYSTEM

A large number of studies demonstrating the crucial role of miRNAs in the development of immune system elements have been conducted in recent years. The miRNA expression profiles of various hematopoietic organs and cell types were shown to be different, with the expression of specific miRNA sets being changed during differentiation of immune system cells. For example, miR-142a, miR-181a, and miR-223 were found to be preferentially expressed in hematopoietic cells [15]. The importance of miRNAs to immune system development was also shown in transgenic mice. Knockout of the *Dicer *gene, which is required for normal maturation of miRNAs, leads to serious disruptions in the development and function of mouse immune system cells and death in the early embryonic period [16]. Today, we know that miRNAs are key regulators of the immune response that affect maturation, proliferation, differentiation, and activation of immune system cells, as well as production of antibodies and release of inflammatory mediators. They are necessary for the normal functioning of both innate and adaptive immunity.

**MiRNAs and regulation of innate immunity**

Innate immunity is the first line of the organism’s defense against infectious agents and the initiator of inflammatory response involving monocytes, macrophages, granulocytes, dendritic cells (DCs), and natural killer (NK) cells.

Monocytes and DCs are able to recognize microbial components through Toll-like receptors (TLRs), triggering a cascade of inflammatory reactions. The activity of Langerhans cells, which are one of the DC subtypes, was shown to be strictly dependent on the Dicer enzyme involved in the formation of mature miRNAs. The cell renewal and apoptosis rate increases in the absence of the Dicer enzyme, which leads to a progressive decrease in the number of Langerhans cells *in vivo *[17]. It was also demonstarted that differentiation of granulocytes in humans is regulated by miR-223 [18], and differentiation of monocytes involves miRNAs belonging to the miR-17-92 and miR-106a-92 clusters [19].

Recognition of the conserved structures of various pathogens by TLR receptors on the surface of DCs and monocytes initiates signal transduction into the cell through a cascade involving important specific kinases IRAK-1, -2, or -4 (interleukin-1 (IL-1) associated kinases) and the tumor necrosis factor receptor-associated factor 6 (TRAF6). This stimulates the release of pro-inflammatory and antiviral cytokines, such as interferon IFN-γ, IFN-β, and the tumor necrosis factor (TNF) [17]. All these stages are also regulated by miRNAs. For example, miR-155, whose synthesis is induced by a number of TLR ligands, is involved in the survival and activation of immune cells through binding to its targets: Src homology-2 domain-containing inositol 5-phosphatase 1 (SHIP1) and suppressor of cytokine signaling 1 (SOCS1), which are negative regulators of the immune response [20]. This leads to stimulation of synthesis of proinflammatory cytokines and, as a consequence, to activation of the adaptive immune response. The* MIR146A *gene expression is immediately induced by a lipopolysaccharide, which is a cell-wall component of Gram-negative bacteria, while miR-146a itself is capable of complementarily binding to 3’-UTR mRNAs of IRAK-1 and TRAF6, inhibiting production of these key signaling proteins, which inhibits activation of the NF-κB factor and reduces production of proinflammatory cytokines IL-6 and TNF [21].

In response to lipopolysaccharides, monocytic cell lines and macrophages also significantly increase expression of miR-132, -125b, -21, and -9, which indicates involvement of miRNAs in controlling the Toll signaling pathway, with some miRNAs (according to the effect pattern) acting at the stage when the organism returns to normal homeostasis after a response to an infection. This feedback regulation is very important, because not only reduced, but also increased activation of the TLR signaling pathway may harm the organism [22].

NK cells provide early protection through disruption of transformed cells and also affect the development of many immune cells, producing various cytokines. Peripheral NK cells not expressing miRNA biogenesis’ genes Dicer or Pasha (*Dgcr8*) had functional disturbances of cellular receptor activation [17], which emphasizes the significance of the miRNA system for the function of NK cells.

All these studies strongly indicate that miRNAs are actively involved in the regulation of the innate immune response.

**MiRNAs and regulation of adaptive immune response**

The adaptive immune response is characterized by specific recognition of foreign antigens by T and B lymphocytes, followed by selection and proliferation of the antigen-specific clones of these cells. This results in both a dramatic increase in the number of T and B lymphocytes responding to an antigen and production of memory cells providing a secondary immune response.

**Fig. 3:**
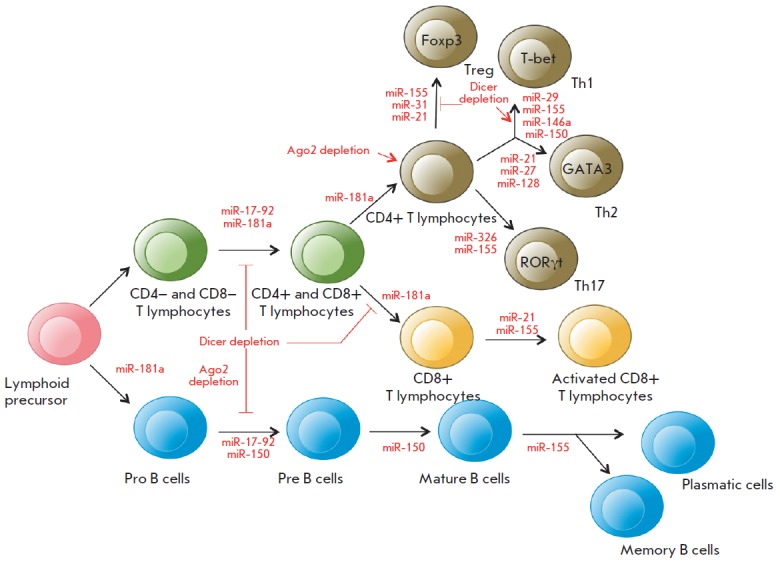
The role of miRNAs in the differentiation of T and B cells (modified from [28]). Th1, Th2, and Th17 are T helper cells; Treg are regulatory T cells; Foxp3, T-bet, GATA3, and RORγt are transcription factors required for the normal development of various T helper cell subsets. See the text for details.

MiRNAs are actively involved in the regulation of the development and differentiation of T and B cells (*Fig. 3*). For example, disruption of miRNA processing in T cells caused by a deletion of the *Dicer *gene reduces the amount of thymocytes and increases their apoptosis at an early developmental stage [23]. The lack of Dicer or AGO2 proteins disrupts differentiation of B cells at different stages and changes the spectrum of secreted antibodies [24, 25]. Furthermore, Dicer deficiency is supposed to affect the program of V(D)J-recombination in developing B cells [25]. At the same time, naive T cells with reduced miRNA expression and production of the AGO2 protein differentiate more rapidly into effector T cells [26].

Many studies have focused on exploring individual miRNAs whose expression is specific at each stage of the differentiation of B and T cells. According to [27], more than 100 miRNAs were identified that may potentially affect the molecular pathways controlling the differentiation and function of innate and adaptive immunity cells. MiR-181a is differentially expressed in hematopoietic cells and is involved in the regulation of differentiation of B and T cells at early developmental stages. Inhibition of miR-181a in immature T cells disrupts both positive and negative selection of T cells, despite the fact that increased expression of this miRNA in mature T cells enhances the sensitivity of their response to an antigen [28]. Along with miR- 181a, miR-17-92 expression is increased in B and T cells precursors and reduced in mature cells, and miR-155 is required for the differentiation of naive T cells into effector cells (Treg, Th1/2, Th17) [29].

A reduction in the amount of Dicer or Drosha in regulatory T cells (Treg) leads to the early development of autoimmune diseases. CD4+ T cells not expressing miRNAs were shown not to be capable of differentiating into Treg cells in the thymus. *MIR155* knockout mice have a reduced amount of Treg cells [30]. At the same time, miR-21 and miR-31 regulate Treg cell differentiation by changing the expression of the basic transcription factor Foxp3 required for the normal development of this subset of CD4+ cells [31]. Unlike Treg cell, a *Dicer *deletion leads to activation of differentiation of naive CD4+ T-cells towards Th1 cells. Increased expression of miR-29 in naive CD4+ T cells inhibits differentiation of Th1 cells and production of IFN-γ. MiR-146a has been demonstrated to be involved in the regulation of differentiation of Th1 cells targeting Traf1 and Irak1 (as previously discussed), as well as Stat1 mRNAs. Furthermore, miR- 146a expression is increased in Th1 cells, whereas this expression is reduced in Th2 cells. An increased expression of miR-21 in T cells promotes *in vitro *differentiation of Th2 cells, while icreased expression of miR-27 and miR-128 reduces production of IL-4 and IL-5 in activated CD4+ T cells. The subset of Th17 cells is regulated by miR-326 that binds to the target* Ets1 *gene and enhances differentiation of these cells and production of IL-17 [31]. At the later stages of differentiation, increased expression of miR-155 and miR-21 in CD8+ cells was observed [32]. MiR-150 prevents the development of mature B cells but promotes activation of T cells through binding to specific transcription factors, including c-Myb and T-bet [33].* MIR155 *knockout mice were detected with disruptions of antibody secretion and switching of the production of antibody isotypes in B cells [34].

Therefore, increasingly growing evidence indicates that miRNAs are involved in the regulation of the immune response. Disruption of this regulation may lead to various pathological conditions, including autoimmune inflammatory processes.

## MiRNAs AND DEVELOPMENT OF AUTOIMMUNE INFLAMMATION

Autoimmune inflammation underlies the pathogenesis of many systemic and organ-specific autoimmune diseases (AIDs), such as systemic lupus erythematosus, MS, rheumatoid arthritis, type 1 diabetes mellitus, autoimmune thyroiditis, Crohn’s disease, etc. The cause of these diseases is considered to be a negative reaction of the immune system to self-tissues that leads to the formation of autoreactive cells and autoantibodies, production of a wide range of pro-inflammatory cytokines and mediators, and eventually to damage and destruction of normal tissues. Now, it is believed that the initial impetus for the development of many AIDs is chronic inflammation that, due to mediators constantly produced by autoimmune cells, exacerbates the negative reaction of the immune system to the self antigens and prevents, through a negative feedback mechanism, immune response completion.

**Fig. 4:**
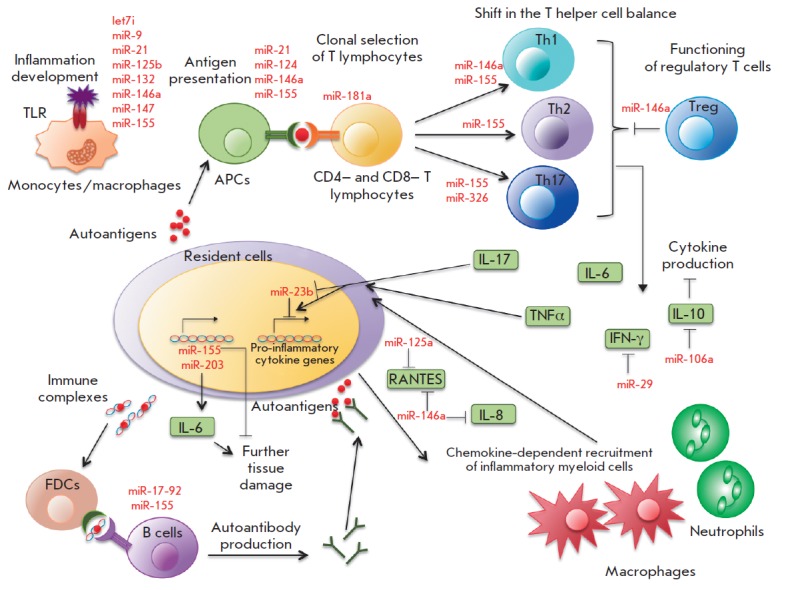
The role of miRNAs in autoimmune inflammation (modified from [35]). APCs are antigen-presenting cells; Th1, Th2, and Th17 are T helper cells; Treg are regulatory T cells; FDCs are follicular dendritic cells. See text for more details.

Analysis of the miRNA profile in AID patients revealed numerous disruptions of miRNA expression [35-37], with the most frequent changes in some miRNAs (e.g., miR-155, -146a, -326, -21, and -181). Specific miRNAs expressed by cells of the immune system and resident cells of tissues can repress the synthesis of key proteins, thereby contributing to the development of the autoimmune inflammatory response at different stages (*Fig. 4*). These stages include an inflammatory reaction; activation of antigen-presenting cells (APCs); recognition of an antigen by specific lymphocyte receptors; differentiation of CD4+ T cells into different subsets; functioning of Treg cells; production of various cytokines; tranduction of the signal into resident cells of various tissues in response to inflammatory cytokines; additional recruitment of inflammatory cells by chemokines and cytokines; formation of germinal centers of B cells and switching of immunoglobulin isotypes; as well as some mechanisms of tissue damage not mediated by immune cells.

As mentioned above, sequential activation of certain miRNAs can control the strength level and duration of the inflammatory response induced by activation of TLR receptors. For example, induction of miR-155 and repression of miR-125b and let-7i caused by activation of TLR receptors were shown to lead to synthesis of various pro-inflammatory cytokines and activation of an adaptive immune response. Induction of expression of miR-146a, -132, and -9, which are negative regulators of inflammation, promotes inhibition of TLR signaling. MiR-21 and miR-147, which are induced later, are involved in the activation of the inflammatory response, inhibiting the synthesis of miR-155 and proinflammatory cytokines [22].

The role of certain miRNAs in the function of APCs and differentiation of CD4+ T cells was described above. Production of several cytokines is directly regulated by miRNAs. For example, miR-29 in T lymphocytes can bind to mRNA of IFN-γ and inhibit its production [30], while miR-23b expressed in resident fibroblast-like synoviocytes can inhibit NF-κB activation by binding to target mRNAs of the *TAB2*, *TAB3*, and *IKK-α *genes in response to inflammatory cytokines [38]. Thus, miRNAs can regulate crosstalk between cytokines produced by immune cells and signal transduction from cytokine receptors to resident cells of various tissues during AID development. MiRNAs can be involved in the recruitment of additional inflammatory cells with participation of chemokines. It was demonstrated that miRNA-125a is involved in the negative regulation of chemokine RANTES (CCL5) production in activated T cells upon development of systemic lupus erythematosus [39], and increased miRNA-146a expression inhibits secretion of chemokines CCL5 and IL-8 in epithelial cells of the human lung [40]. Immunoglobulin isotype switching can also be disrupted in the absence of some miRNAs; e.g., miR-155 [34]. Finally, activation of certain matrix metalloproteinases promoting penetration of immune cells into the inflammatory lesion was shown to be also regulated by miRNAs [41].

A screening comparative study of miRNA expression in resident cells in inflammatory lesions in patients with rheumatoid arthritis, systemic lupus erythematosus, and also in animal models of these diseases and MS revealed miRNA expression disturbances common to these autoimmune diseases: expression of miR-23b and miR-30a-5p was enhanced, and expression of miR-214 and miR-146a was reduced [38].

The accumulated data suggest a significant role of miRNAs in the development of AIDs. Let us consider this role in more details using the example of multiple sclerosis, which is one of the most studied AIDs.

## THE ROLE OF miRNAs IN THE DEVELOPMENT OF MULTIPLE SCLEROSIS

MS is a severe chronic autoimmune inflammatory CNS disease associated with a complex of immune-mediated pathological reactions that destruct the myelin sheath of neurons, which eventually leads to irreversible loss of neurological functions and severe disability.

Currently, the etiology and pathogenesis of MS are not fully elucidated; however, numerous studies suggest the triggering role of the autoimmune process causing damage to the myelin sheath of nerve cells in the CNS. Activation of anergic T and B lymphocytes in the periphery (outside the CNS) is the first stage in MS immunopathogenesis. MS is associated with disturbance of both cellular and humoral immunity. The development of MS is characterized by shifting of the balance of CD4+ T helper cells towards Th1 and Th17 subsets and also by dysfunction of Treg cells. Disruption of the blood-brain barrier permeability promotes penetration of autoreactive cells into the CNS, where they are reactivated by proteins and lipids of the neuron myelin sheath. Then, myelin-specific cells are involved in the development of pathologic demyelinating lesions (plaques). Activated resident CNS cells (microglia and astrocytes) also participate in the formation of the plaques. They produce cytokines and chemokines, mainly proinflammatory, that additionally recruit both autoreactive T and B lymphocytes and monocytes/ macrophages to demyelinating plaques; these cells, in turn, secrete a variety of the active molecules (cytokines, antibodies, oxygen and nitrogen radicals, proteases) involved in further damage to the myelin sheath and oligodendrocytes. Long-term and severe demyelination causes axonal death that leads to the development of symptoms of a persistent neurological deficit. Damage to oligodendrocytes and myelin is accompanied by the release of a large number of autoantigens providing impetus to the further development of the autoimmune process.

**MiRNAs and the autoimmune inflammatory process in experimental autoimmune encephalomyelitis**

The relationship between immunopathological and neurodegenerative processes enables use of experimental autoimmune encephalomyelitis (EAE) as the primary animal model for studying the role of miRNAs in MS development.

One of the first studies demonstrated that *MIR155* knockout mice are resistant to EAE due to reduced differentiation of Th1 and Th17 cells upon autoimmune inflammation [42]. Screening studies revealed changes in the expression of many miRNAs upon EAE. For example, 43 miRNAs were identified whose expression in the lymph nodes of EAE rats was higher than that in rats resistant to EAE [43]; 33 of these miRNAs were previously associated with the development of MS and other AIDs. In oligodendrocytes of EAE mice, expression of 56 miRNAs was lower than that in oligodendrocytes of normal mice; the lowest expression level was that of miR-15a-5p, -15b-5p, -20b-5p, -106b-5p, -181a- 5p, -181c-5p, -181d-5p, -320-3p, -328-3p, and -338-3p [44].

Studying the role of miRNAs in the EAE development helped identify specific target genes of some miRNAs and evaluate their involvment in the pathogenesis of the disease (*Table 1*). The main method to induce EAE in C57Bl/6 [38, 42, 47-57] and SJL [45] mice or Dark Agouti and PVG rats was immunization with the myelin oligodendrocyte glycoprotein, proteolipid protein, or their immunogenic peptides in a complete Freund’s adjuvant in combination with the pertussis toxin [43]. The studies were performed mainly in cells of the immune system (in particular, CD4+ T lymphocytes) and also in various cells of the nervous tissue. An increase in the expression level of miRNA genes (except miR-20b and miR-132/212) was mostly observed in CD4+ T cells; in nervous system cells, expression of the three miRNAs was reduced, and that of two miRNAs was enhanced. The main targets of miRNAs both in CD4+ T lymphocytes and in nervous system cells were mRNAs of genes of transcription factors and modulators of transcription factor activity and genes of signaling pathway elements and cytokines. It is important to note that targets of miR-29b and miR-20b are mRNAs of the *TBX21 *and *RORC *genes encoding T-bet and RORγt, which are the main transcription factors involved in the differentiation of Th0 cells to Th1 and Th17 cells, respectively. The target of miR-326 is the* ETS1 *gene encoding a transcription factor that directly controls the expression of cytokine and chemokine genes and is involved in the regulation of differentiation and proliferation of lymphoid cells.

**Table 1:** Targets and the possible mechanisms of the effect of certain miRNAs whose expression was disrupted during the development of experimental autoimmune encephalomyelitis in mice.

miRNA	Cell type	Change in expression*	Target genes	Effect of changed miRNA expression	Reference
let-7e	CD4+ T lymphocytes	↑	IL10	Stimulation of development of Th1 and Th17 cells	[48]
miR-17	CD4+ T lymphocytes	↑	IKZF4	Increased polarization of Th17 cells	[49]
miR-19b	CD4+ T lymphocytes	↑	PTEN	Activation of Th17 cell differentiation	[49]
miR-20b	CD4+ T lymphocytes	↓	RORC, STAT3	Activation of Th17 cell differentiation	[50]
mir-21	CD4+ T lymphocytes	↑	SMAD7	Activation of Th17 cell differentiation	[51]
miR-23b	Spinal cord cells	↓	TAB2, TAB3, CHUK	Stimulation of IL-17-mediated autoimmune inflammation	[38]
miR-26a	Brain cells	↓	IL6	Increased expression of Th17-mediated cytokines	[52]
miR-29b	CD4+ T lymphocytes	↑	TBX21, IFNG	Regulation of Th1 cell differentiation	[53]
miR-124	Bone marrow macrophages	↓	CEBPA, SPI1	Activation of phagocytic activity, inhibition of microglia differentiation	[47]
miR- 132/212	CD4+ T lymphocytes	↓	ACHE	Stimulation of T cell proliferation and production of IL-17 and IFN-γ; increased catalytic activity of acetylcholinesterase	[54]
miR-146a	Bone marrow stem cells	↑	PTGES2	Inhibition of prostaglandin E2 synthesis	[45]
miR-155	CD4+ T lymphocytes	↑	SOCS1	Stimulation of development of Th1 and Th17 cells	[42]
		↑	INPP5D	Disturbance of myelin proliferation	[42]
miR-301a	CD4+ T lymphocytes	↑	PIAS3	Regulation of Th17 cell differentiation	[55]
miR-326	CD4+ T lymphocytes	↑	ETS1	Stimulation of development and proliferation of Th17 cells	[56]
miR-873	Primary astrocyte culture	↑	TNFAIP3	Stimulation of production of inflammatory cytokines and increased demyelination of nerve fibers	[57]

*Hereinafter: an increase (↑) or decrease (↓) in miRNA expression upon experimental autoimmune encephalomyelitis.

Targets of other miRNAs include genes of members of various signaling pathways, in particular the NFkB (*TNFAIP3 *and *CHUK*) and JAK/STAT (*STAT3*,* SMAD7*, *SOCS1*, and *PIAS3*) pathways, as well as genes encoding phosphatases (*INPP5D *and *PTEN*). Genes of some cytokines (*IL-10*, *IL-6*, and *IFNG*) and signaling pathways of IL-1 and IL-17 cytokines (*TAB2* and *TAB3*) are also targets of miRNAs, whose expression varies upon EAE.

The action of miRNAs (such as let-7e, miR-155, -17- 92, -20b, -21, -29b, -301a, and -326) on the target is mainly observed in the disruption of differentiation and proliferation of Th1 and Th17 cells, which are believed to play a major role in the EAE development. MiR-26a and miR-873 stimulate production of pro-inflammatory cytokines, affecting the neuroinflammatory process and severity of EAE. In addition, miR-155 is involved in the disruption of myelin proliferation, which may also contribute to the development of neurodegenerative processes. An increased level of miR-146a in neuronally- differentiated bone-marrow-derived mesenchymal stem cells (BMSCs) during EAE inhibits the synthesis of prostaglandin E2, which may lead to increased production of TNF and IFN-γ by activated DC and T cells [45, 46]. Reduced miR-124 expression promotes activation of the phagocytic activity and inhibition of microglial differentiation, which leads to worsening of EAE in animals [47].

Thus, EAE proved to be an adequate experimental model suitable for studying the differential expression of miRNAs in autoimmune inflammation and for identifying the role of individual miRNAs in regulation of differentiation of Th1 and Th17 cells and synthesis of pro- and anti-inflammatory cytokines.

**MiRNAs and the development of autoimmune inflammation in MS**

**Table 2:** MiRNAs whose expression is altered in multiple sclerosis.

miRNA source	Multiple sclerosis form	miRNAs differentially expressed in MS patients compared to a control	Change in expression	Reference
Whole blood	RRMS	miR-142-3p, -145, -186, -223, -442a, -491-5p, -584, -664, -1275	↑	[58]
		miR-20b	↓	
	RRMS, CIS	miR-16-2-3p, -574-5p	↑	[59]
		miR-7-1-3p, **-20a-5p, -20b**, -146b-5p, -3653	↓	
	RRMS, PPMS, SPMS	miR-17, -20	↓	[60]
MNCs	RRMS	miR-326	↑	[56]
		miR-18b, -193a, -328, -599	↑	[61]
		let-7d, miR-145, -744	↑	[62]
		miR-142-3p, **-146a, -155, -326**	↑	[63]
	RRMS, PPMS, SPMS	let-7g, miR-150	↓	[64]
	RRMS, CIS	**miR-29a-3p, -29c-3p**, -532-5p	↓	[65]
CD4+ T lymphocytes	RRMS	miR-326	↑	[56]
		**miR-17-5p**, -193a, -376a, -485-3p	↑	[66]
		miR-34a, -126, -497	↓	
	RRMS, PPMS, SPMS	miR-27b, -128, -340	↑	[67]
		miR-29b	↑	[53]
CD4+ CD25+ T regulatory lymphocytes	RRMS	**miR-19a, -19b**, -25, -93, -106b	↑	[68]
B lymphocytes	RRMS	**miR-19b**, -106b, -191, -551a	↑	[69]
Plasma	N/A	miR-22, -422a, -572, -614, -648, -1826	↑	[70]
		miR-1979	↓	
CSF*	RRMS, PPMS, SPMS	miR-181c, -633	↑	[71]
		miR-922	↓	
Demyelinating plaque**	RRMS, PPMS, SPMS	**miR-21, -23a**, -27a, -34a, -142-3p, **-146a, -155**, -199a, **-326**, -346, -650	↑	[72]

*The cerebrospinal fluid of patients with other neurological diseases was used as a control.

**Postmortem sections of brain white matter obtained from patients without a neurologic disease were used as a control.

Note. SPMS – secondary progressive multiple sclerosis (MS); CIS – clinically isolated syndrome; MNCs – mononuclear cells; PPMS – primary progressive MS; RRMS – relapsing remitting MS; CSF – cerebrospinal fluid. MiRNAs whose expression is changed both in multiple sclerosis and in experimental autoimmune encephalomyelitis are shown in bold.

*Table 2* presents the results of a study of miRNA expression in MS patients. Various tissues and cells were used to isolate miRNAs: blood components, cerebrospinal fluid, and demyelinating plaques. miRNA expression levels were compared between a control group of healthy people and patients with various MS forms.

The group of MS patients consisted mostly of patients with relapsing-remitting MS (RRMS), clinically isolated syndrome, primary progressive MS, and secondary progressive MS. *Table 2* shows that the spectrum of expressed miRNAs is very broad and probably depends on the source of their isolation and/or the MS form.

Changes in the expression of certain miRNAs were observed in various cells. For example, expression of miR-142-3p, -155, and -326 was increased both in demyelinating plaques and in the whole blood of MS patients; expression of miR-19b and miR-106b was increased in Treg and B cells; and expression of miR-145 was increased in the whole blood and peripheral blood mononuclear cells. Opposite effects, depending on the cell type, were observed for certain miRNAs (miR-17, miR-34a): expression of miR-17 was increased in the whole blood and reduced in CD4+ T cells; expression of miR-34a was increased in plaques and decreased in CD4+ T cells. A number of changes (shown in bold in* Table 2*) in the expression of miRNAs (miR-17, -19, -20, -21, -23, -29, -146, -155, -326) coincided with the data obtained in EAE models. However, different miRNA expression patterns were observed in many cases that may be explained by various causes, including experimantal conditions. It should be noted that the data on miRNA expression levels presented in *Table 2*are not correlated with a MS stage and the approach to the treatment of MS patients.

**Table 3:** Target mRNAs and the possible mechanisms of the effect of certain miRNAs whose expression is changed during the development of multiple sclerosis in humans.

miRNA	Tested cells	Change in expression	Target genes	Putative functions	Reference
miR-17	CD4+ T lymphocytes	↑	TGFBR2, PTEN, BCL2L11, CDKN1A	Proliferation and activation of T cells	[73]
miR-34a miR-155 miR-346	Demyelinating plaques	↑	CD47	Stimulation of myelin phagocytosis	[72]
miR-132	B lymphocytes	↑	SIRT1	Increased production of pro-inflammatory cytokines	[74]
miR-320a	B lymphocytes	↓	MMP9	Disturbance of HEB permeability	[75]
miR-340	CD4+ T lymphocytes	↑	IL4	Shift of the balance of Th2/Th1 cytokines towards Th1 cytokines	[67]

Target genes of some miRNAs, whose expression is changed during MS development, were identified in B and CD4+ T cells. These genes and their putative functions are shown in *Table 3*. The gene encoding the cell adhesion molecule SD47 is the target of miR-34a, miR-155, and miR-346. Other targets are genes encoding regulators of apoptosis (BIM) and transcription (SIRT1), cell proliferation inhibitor (p21), matrix metalloprotease 9 (MMP9), and cytokine IL-4. The action of miRNAs on these targets causes stimulation of myelin phagocytosis, changes in the blood brain barrier permeability, and disturbance of proliferation and activation of T cells and secretion of pro- and anti-inflammatory cytokines.

The analysis of the data presented in *Tables 2 *and *3* indicates a variety of miRNA differential expression profiles in MS, depending on the type of analyzed cells. Given the complex nature of MS, it may be assumed that miRNA expression determines the stages of the clinical course of MS; however, the available data are not sufficient to draw final conclusions. Further investigation of miRNA differential expression may help to identify potential MS biomarkers and clinical MS patterns, as well as shed light on the mechanisms of miRNA action. Of particular interest are the trigger mechanisms underlying the processes that control the transition of patients from remission to relapse and from relapse to remission upon a RRMS clinical course.

In general, investigation of the role of miRNA in the epigenetic regulation of autoimmune inflammation in various inflammatory AIDs may not only facilitate the understanding of the processes that maintain the stability and plasticity of the immune system, but also affect the development of strategies for the prevention and treatment of these serious social diseases.

## Abbreviations

AID: autoimmune disease
APC: antigen-presenting cell
SPMS: secondary progressive multiple sclerosis
DCs: dendritic cells
PBMCs: peripheral blood mononuclear cells
RRMS: relapsing-remitting multiple sclerosis
MS: multiple sclerosis
TF: transcription factor
CNS: central nervous system
EAE: experimental autoimmune encephalomyelitis
NKs: natural killer cells
Th1/2/17: T-helper 1/2/17 cells
TLR: Toll-like receptor
Treg: regulatory T cells
